# Asthma hospitalisations and heat exposure in England: a case–crossover study during 2002–2019

**DOI:** 10.1136/thorax-2022-219901

**Published:** 2023-04-17

**Authors:** Garyfallos Konstantinoudis, Cosetta Minelli, Holly Ching Yu Lam, Elaine Fuertes, Joan Ballester, Bethan Davies, Ana Maria Vicedo-Cabrera, Antonio Gasparrini, Marta Blangiardo

**Affiliations:** 1 MRC Centre for Environment and Health, Department of Epidemiology and Biostatistics, School of Public Health, Imperial College London, London, UK; 2 National Heart and Lung Institute, Imperial College London, London, UK; 3 UK Health Security Agency, London, UK; 4 Climate and Health Program (CLIMA), Barcelona Institute for Global Health (ISGlobal), Barcelona, Spain; 5 UK Small Area Health Statistics Unit, imperial College London, London, UK; 6 Institute of Social and Preventive Medicine, University of Bern, Bern, Switzerland; 7 Oeschger Center for Climate Change Research, University of Bern, Bern, Switzerland; 8 Department of Public Health Environments and Society, London School of Hygiene Tropical Medicine, London, UK; 9 Centre for Statistical Methodology, London School of Hygiene Tropical Medicine, London, UK; 10 Centre on Climate Change and Planetary Health, London School of Hygiene Tropical Medicine, London, UK; 11 School of Public Health, Imperial College London, London, UK

**Keywords:** asthma epidemiology

## Abstract

**Background:**

Previous studies have reported an association between warm temperature and asthma hospitalisation. They have reported different sex-related and age-related vulnerabilities; nevertheless, little is known about how this effect has changed over time and how it varies in space. This study aims to evaluate the association between asthma hospitalisation and warm temperature and investigate vulnerabilities by age, sex, time and space.

**Methods:**

We retrieved individual-level data on summer asthma hospitalisation at high temporal (daily) and spatial (postcodes) resolutions during 2002–2019 in England from the NHS Digital. Daily mean temperature at 1 km×1 km resolution was retrieved from the UK Met Office. We focused on lag 0–3 days. We employed a case–crossover study design and fitted Bayesian hierarchical Poisson models accounting for possible confounders (rainfall, relative humidity, wind speed and national holidays).

**Results:**

After accounting for confounding, we found an increase of 1.11% (95% credible interval: 0.88% to 1.34%) in the asthma hospitalisation risk for every 1°C increase in the ambient summer temperature. The effect was highest for males aged 16–64 (2.10%, 1.59% to 2.61%) and during the early years of our analysis. We also found evidence of a decreasing linear trend of the effect over time. Populations in Yorkshire and the Humber and East and West Midlands were the most vulnerable.

**Conclusion:**

This study provides evidence of an association between warm temperature and hospital admission for asthma. The effect has decreased over time with potential explanations including temporal differences in patterns of heat exposure, adaptive mechanisms, asthma management, lifestyle, comorbidities and occupation.

**WHAT IS ALREADY KNOWN ON THIS TOPIC**

- Heat exposure has been reported to increase risk of asthma hospital admissions.

**WHAT THIS STUDY ADDS**

- We assess the effect of warm temperatures on asthma hospitalisation using 18 years’ worth of individual-level nationwide data in England. We examine vulnerabilities by age, sex, time and space and find an increased risk in males aged 16–64 (2.10%, 1.59% to 2.61%) and the early years of the study (2.96%, 2.56% to 3.37%) and the regions of Yorkshire and the Humber and West and East Midlands.

**HOW THIS STUDY MIGHT AFFECT RESEARCH, PRACTICE OR POLICY**

- This study is the first to provide evidence that the effect of heat exposure in patients with asthma has attenuated over time. Potential reasons for this include temporal adaptation, differences in lifestyle, comorbidities and occupation.

## Introduction

Asthma is the second most prevalent chronic respiratory disease globally (3.6%) and the first in the Western world.^1 2^ The prevalence of asthma in the UK is among the highest in the world with direct National Health Service (NHS) costs for managing asthma being estimated at £1 billion per annum with 12% of these costs being for hospital care.^3 4^ Several risk factors, such as smoking, physical activity, medication, etc, have been known to trigger asthma symptoms.^5^ In addition, environmental risk factors such as inhaling cold air, air pollutants and allergens can trigger asthma symptoms and exacerbations.^5–7^ Previous studies have examined the role of warm temperatures on asthma hospitalisation, with the results being inconclusive.^8–16^

The impact of ambient warm temperature on asthma hospitalisation has received considerable attention recently.^8–16^ Studies in Korea, Shanghai, China and the Los Angeles County, USA reported weak, if any, evidence of an association between increasing mean daily or maximum temperature and asthma hospitalisations.^8 10 11^ In contrast, studies in Hong Kong, Himeji City, Japan, Taiwan, 1816 cities in Brazil, Maryland, USA, New York City, USA and Beijing, China reported an increased asthma hospitalisation risk with higher temperature especially during summer months.^11 13 15–19^

Some of the reasons contributing towards the above-mentioned discrepancies include decisions about the lags, temperature metrics, outcome definitions and selection of confounders, in addition to different adaptation mechanisms (eg, prevalence of air conditioning, building infrastructure, etc), population characteristics (eg, deprivation, age distribution, etc) and meteorology across the different countries. Methodological aspects might also limit the generalisability of previous studies. The temporal or geographical resolution in some of the previous studies is coarse leading to a less accurate exposure assignment; most studies examine daily hospitalisation with one study considering monthly hospitalisations,^14^ and most previous studies assign exposure at the city level.^15–17^ Most studies with individual data were conducted in urban settings, which limits the generalisability of their results given that temperatures in urban settings are typically higher and less variable.^15–17^ One study was nationwide in Taiwan, but the geographical resolution was coarse and the exposure available only at 25 meteorological stations.^11^ Previous studies have examined effect modification by age and sex,^15 17^ space^8 11 16^ and by time,^16^ but none considered all these dimensions together.

In this nationwide study in England, we examine the effect of ambient temperature on hospital admissions for asthma during 2002–2019. This study aims to address some of the limitations in previous studies by covering one of the largest temporal windows for asthma hospitalisations in the literature, using individual data and exploiting high geographical resolution to link outcome (~100 m) with the exposure (1 km). We account for different meteorological confounders and investigate how the effect of ambient temperature on asthma hospitalisations is modified by age and sex. We also examine how the effect is modified by time and region, as we expect the effect to have attenuated over time, for reasons including adaptation (including changes in behaviours, heat-health warnings, etc), improvement in asthma treatment and awareness of asthma management, etc, and to be different across space (due to factors that might also vary in space such as differences in green space, socioeconomic deprivation, etc).^20 21^

## Methods

### Study population

We retrieved all inpatient hospital admissions for asthma in England during 2002–2019 from NHS Digital Hospital Episode Statistics data held by the UK Small Area Health Statistics Unit. Age, postcode of residence at time of hospitalisation and date of hospitalisation were available for each record. We investigated the main diagnostic groups J45 (Asthma) and J46 (Status asthmaticus) according to the International Classification of Diseases version 10 as the main diagnosis of the hospitalisation.^22^ The analysis is restricted to June, July and August during which the highest temperatures are reached in England. Because of considerations related with the diagnostic uncertainty and miscoding of asthma in young children,^23^ we excluded the 0–4 age group from our analysis.

### Exposure

Daily temperatures at 1 km*×*1 km resolution were available from the UK Met Office and obtained with methods described elsewhere.^24^ In brief, the daily temperature in each grid is estimated based on inverse distance weighted interpolation of monitoring data, also accounting for latitude and longitude, elevation, coastal influence and proportion of urban land use. We calculated mean daily temperature taking the average of maximum daily and minimum daily temperatures during 2002–2009. We selected the mean as the metric to capture the average daily exposure to temperature, in contrast with the min, which reflects exposure at night, or the max, which reflects exposure at midday. To assign daily mean temperature to health records, the postcode centroids of each patient were spatially linked to the 1 km*×*1 km grid cell, applying a 100 m fuzziness to the postcode location to fulfil governance requirements.^20^ For the main analysis, we averaged the daily mean temperatures across the 3 days prior to the hospital admission (lags 0–3), as the delayed effect of heat exposure on health outcomes is expected to last a few days.^15 20 25^

### Confounders

Our main assumptions about possible confounders are shown on the directed acyclic graph (DAG) in online supplemental figure S1. Notice that confounders accounted for through the study design, for instance, deprivation, are omitted from the DAG. Based on the DAG, we decided to account for meteorology (relative humidity, precipitation and wind speed) and national holidays, whereas we assumed that air pollution species (eg, NO_2_, PM_2.5_, PM_10_ and O_3_) and grass pollen are more likely to mediate, rather than confound, the relationship between temperature and asthma hospitalisation risk.^26^ Mean daily precipitation (mm) at 1 km*×*1 km resolution between 2002 and 2019 was retrieved from the UK Met Office.^24^ Wind speed and relative humidity were retrieved from the UERRA regional reanalysis for Europe and were available daily at 11 km×11 km spatial resolution.^27^ National holidays were defined as a binary variable, 0 being a holiday and 1 otherwise. We accounted for national holidays as hospital admission rates are different during a national holiday and at the same time exposure to temperature might be greater as more people are outdoors.^28^

10.1136/thorax-2022-219901.supp1 Supplementary data

### Effect modification

We examined effect modification by age group, sex (males, females), period (2002–2007, 2008–2013 and 2014–2019) and space (nine regions in England, online supplemental figure S2). The subgroup analysis by age group was done for children (5–15), working age adults (16–64) and older adults (65+), selected for consistency with previous studies.^15 17^

### Statistical methods

We used a time-stratified case–crossover design, commonly used for analysing the effect of transient exposures.^29–31^ The temperature on the day of the asthma hospitalisation (event day) is compared with the temperature on non-event days. Within this design, each case acts as its own control, controlling for individual-level factors that do not vary over time (eg, age, sex and ethnicity) or vary slowly (eg, deprivation). We selected non-event days on the same day of the week, calendar month and year as the event day to avoid overlap bias,^32^ which also accounts for seasonality (within summer) and long-term trends.^33^

We specified Bayesian hierarchical conditional Poisson models, with a fixed effect on the event/non-event day grouping.^33 34^ The above specification offers an alternative to the conditional logistic model allowing for more flexibility and reducing the computational burden.^34^ We accounted for patient clustering due to recurrent hospitalisations using a parameter term per patient with exacerbation history, specified as a realisation from a normal distribution with zero mean and common variance. To investigate effect modification, we repeated this analysis by age, sex, period and region.

### Sensitivity analyses

As the effect of temperature on health is typically non-linear,^20 25^ we investigated departures from the linearity assumption. To do so, we modelled temperature using a random walk of order 2, a Gaussian process over the temperature domain with zero mean and covariance that depends on the two previous and two subsequent observations.^35^ We also examined sensitivity with respect to the lag choice, focusing on any lag between 0 and 5.

All results are reported as medians and 95% credible intervals (CrI; 95% probability that the true values lie within this interval) of % increase in hospitalisation risk for every 1°C increase in temperature across the unadjusted and fully adjusted (for rainfall, relative humidity, wind speed and national holidays) models. All analyses are run in Integrated Nested Laplace Approximation.^36^ The code for running the analysis is available online at https://github.com/gkonstantinoudis/asthma_temperature.

### Post hoc analysis

In a post hoc analysis we examined how the effect of temperature varies by year. We first fitted the fully adjusted model for each age group and year separately. We then drew 1000 from the posteriors of the effect of temperature for each age group and year and fitted 1000 linear models by age group to examine the temporal trend of the temperature effect.

## Results

### Population

We retrieved 1 268 725 records with a hospital admission for asthma during 2002–2019 in England. After removing 6116 duplicate records, 12 records with place of residence outside England, 1 002 512 that did not occur during summer months, 34 605 aged <5 years and 180 records with missing sex or age, we had 260 085 records available for the analysis (online supplemental figure S3). Most of the admissions occurred for people between 16 and 64 years old (n=135 011) and females (n=81 336) (online supplemental table S1).

### Exposure

**Figure 1:**
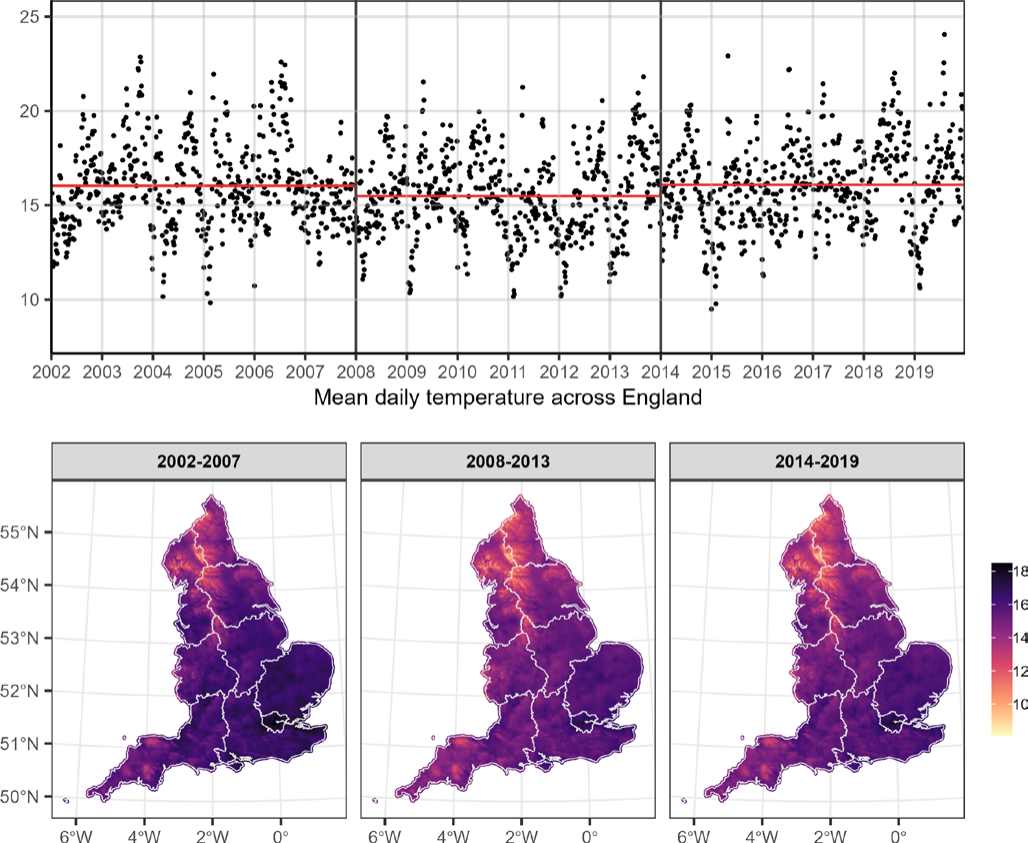
Top panel: mean of the mean daily temperature (^o^C) during summer months across England between 2002 and 2019. The red lines show the mean across the different periods (2002–2007, 2008–2013 and 2014–2019). Bottom panel: mean of the mean daily summer temperature (^o^C) across the 1 km×1 km grid by period. The grey areas define the regions in England.

Figure 1 shows the mean of the mean daily temperature across all 1 km×1 km grid cells (top panel) and across the summer months by period (2002–2007, 2008–2013 and 2014–2019) in England. The maximum mean daily temperature across England is 24.1°C and was observed in 2019 (top panel, figure 1). The mean of the mean daily temperature across England was 16.0°C during 2002–2007, 15.5°C during 2008–2013 and 16.1°C during 2014–2019 (red lines, top panel, figure 1). The mean of the mean daily temperature across the summer months varied from 8.9°C to 18.5°C during 2002–2007, from 8.1°C to 17.5°C during 2008–2013 and from 8.0°C to 17.8°C during 2014–2019 (bottom panel, figure 1).

### Age and sex effect modification

**Figure 2:**
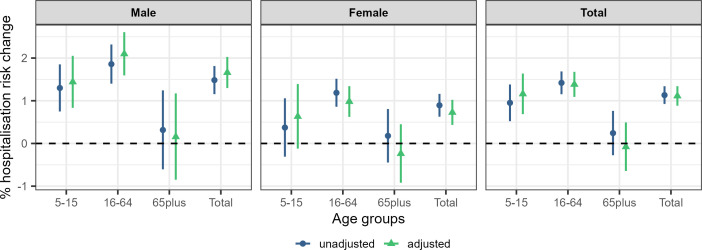
Median percentage asthma hospitalisation risk for every 1°C increase in the daily mean summer temperature and 95% credible intervals by sex and age for the unadjusted and fully adjusted (precipitation, relative humidity, wind speed, national holidays and recurrent hospitalisations) models.

Figure 2 shows the % change in hospitalisation risk for every 1°C increase in the ambient summer temperature for the different age and sex subgroups during 2002–2019 across the unadjusted and the adjusted models. Accounting for rainfall, relative humidity, wind speed and national holidays does not seem to affect much the observed relationship (figure 2). Overall, we found a 1.13% (0.92% to 1.34%) and 1.11% (0.88% to 1.34%) increase in the risk of hospital admission for every 1°C increase in the ambient summer temperature in the unadjusted and adjusted models, respectively (online supplemental table S2). The effect is consistently higher for males and there is weak, if any, evidence of an effect of temperature for people aged 65 or older. The highest effect was observed for males aged 16–64 with a 1.86% (1.40% to 2.32%) and 2.10% (1.59% to 2.61%) increase in the hospitalisation risk in the unadjusted and adjusted models (figure 2 and online supplemental table S2).

### Temporal effect modification

**Figure 3:**
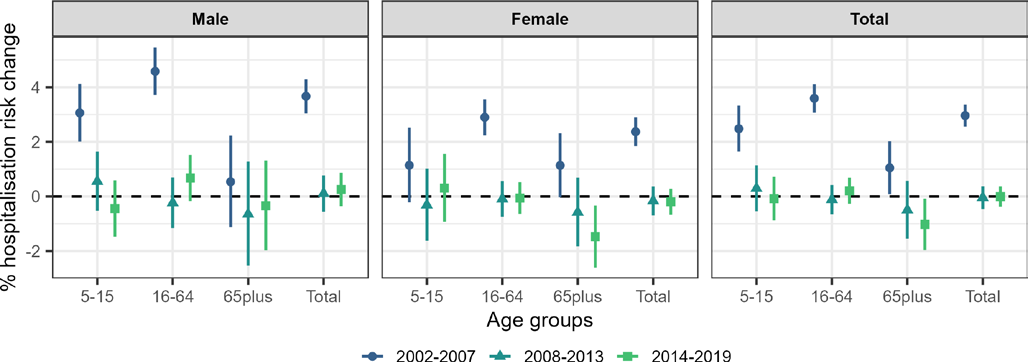
Median percentage asthma hospitalisation risk for every 1°C increase in the daily mean summer temperature and 95% credible intervals by sex, age and period (2002–2007, 2008–2013 and 2014–2019) for the fully adjusted (precipitation, relative humidity, wind speed, national holidays and recurrent hospitalisations) models.

We examined temporal vulnerabilities of asthma hospitalisation to temperature during summer months. Using the adjusted models, we observe an association of temperature with asthma hospitalisation during 2002–2007, with a 2.96% (2.56% to 337%) increase in hospitalisation risk per 1°C increase in temperature, whereas there is inconclusive evidence for the periods 2008–2013 (−0.04%; −0.46% to 0.37%) and 2014–2019 (−0.01%; −0.39% to 0.37%) (figure 3 and online supplemental table S2). The total effect in figure 2 seems to be mainly driven by males, 16–64 years old, during 2002–2007, with a 4.58% (3.71%, 5.46%) increase in the hospitalisation risk for every 1°C increase in temperature (figure 3 and online supplemental table S3). For the rest of the age and sex groups assessed by period the evidence is weaker (figure 3 and online supplemental table S2).

### Spatial effect modification

**Figure 4:**
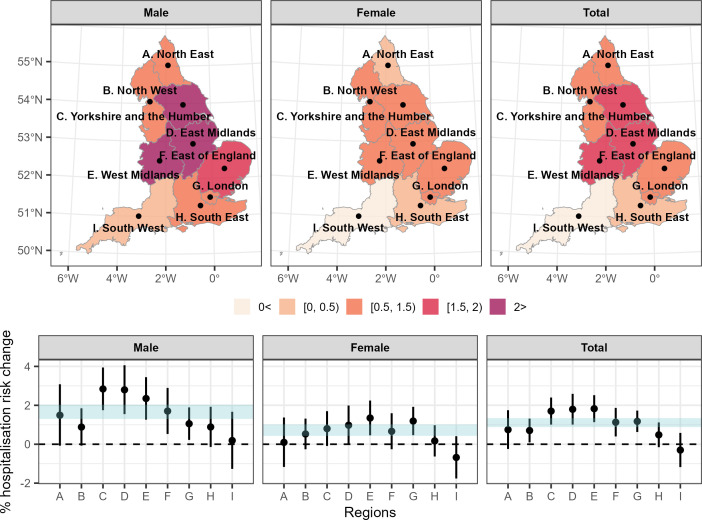
Top panel: median percentage asthma hospitalisation risk for every 1°C increase in the daily mean summer temperature by sex and region based on the fully adjusted (precipitation, relative humidity, wind speed, national holidays and recurrent hospitalisations) models. Bottom panel: median percentage asthma hospitalisation risk for every 1°C increase in the daily mean summer temperature and 95% credible intervals (CrI) by region based on the fully adjusted (precipitation, relative humidity, wind speed, national holidays and recurrent hospitalisations) models. The shaded area illustrates the 95% CrI of the asthma hospitalisation risk nationwide by sex (fully adjusted models).

Figure 4 presents the spatial vulnerabilities by region and by sex in the adjusted models. We observe that males in Yorkshire and the Humber and East and West Midlands are the most vulnerable with a % increase in hospitalisation risk being larger than 2% per 1°C increase in temperature in the adjusted models (figure 4). Populations in the South West are consistently the least vulnerable (figure 4).

### Sensitivity analyses

Allowing for flexible fits using random walk of order 2 to model temperature validated our linearity assumption in general (online supplemental figure S4). The subgroup deviating from linearity is females between 15 and 65, suggesting an effect most prominent for temperatures higher than 20°C (online supplemental figure S4). When we examined the different lags we found that the lags contributing most to the effect are 0–3 (online supplemental figure S5).

### Post hoc analysis

**Figure 5:**
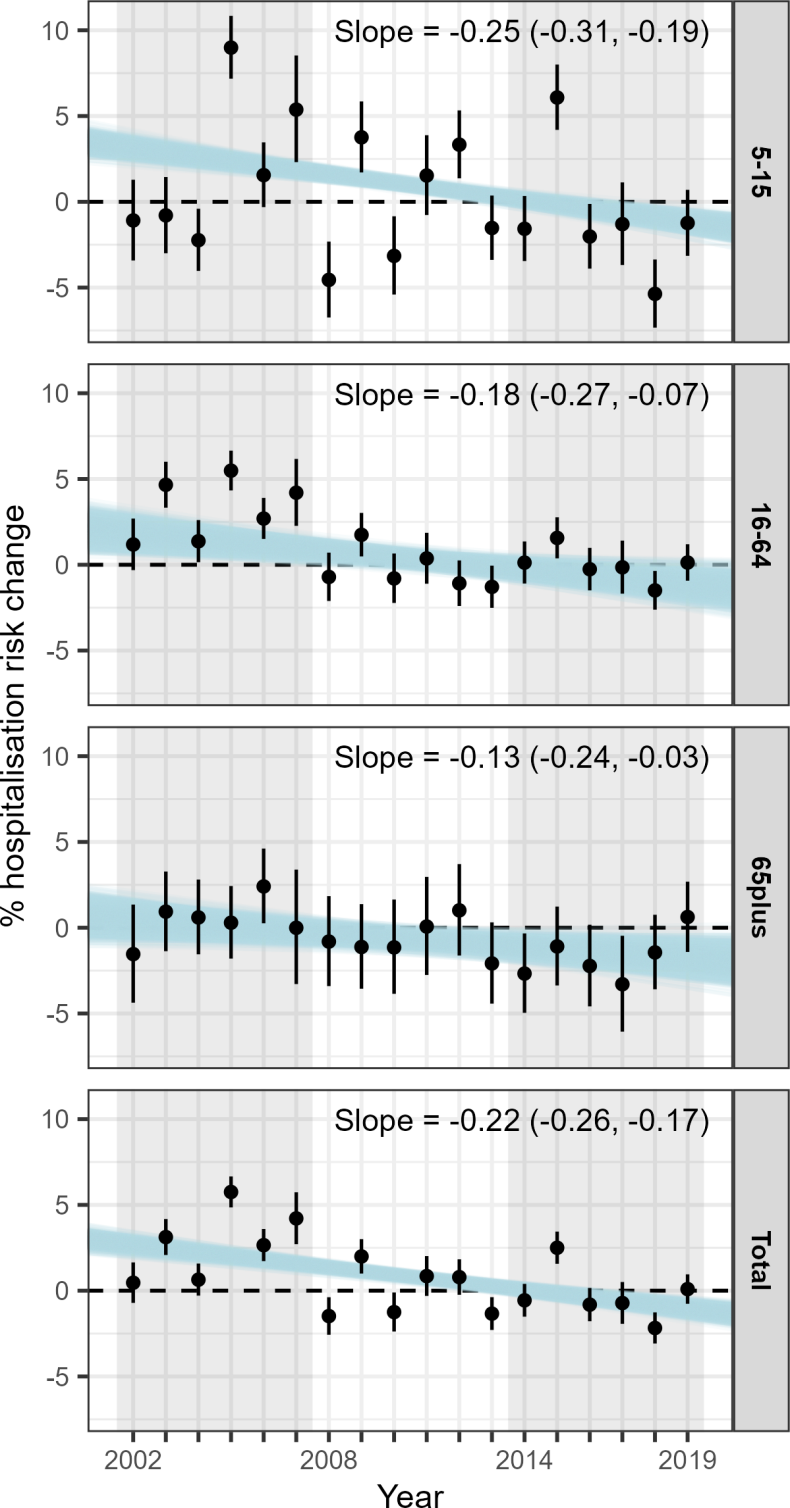
Median percentage asthma hospitalisation risk for every 1°C increase in the daily mean summer temperature and 95% credible intervals by age and year for the fully adjusted (precipitation, relative humidity, wind speed, national holidays and recurrent hospitalisations) models. The blue lines reflect the uncertainty of linear temporal trend of the effect. The text annotation gives the median of the slopes together with the 95% credible intervals. The grey shading on the background defines the three periods (2002–2007, 2008–2013, 2014–2019).

The results of the post hoc analysis are shown in figure 5. The 1000 blue lines per age group show an overall decreasing trend on the temperature effect of the years, varying from −0.25 (−0.31, 0.19) in people aged between 5 and 15 years to −0.08 (−0.13, –0.24) in people aged over 65 years (figure 5). For the total age group, the effect is −0.22 (−0.27, –0.17), suggesting that the percentage asthma hospitalisation risk per 1°C increase in temperature is decreasing by 0.22 for each coming year (figure 5).

## Discussion

In this nationwide study in England investigating the short-term effect of ambient summer temperature on asthma hospitalisations during 2002–2019, we find evidence of an increased risk of asthma hospitalisation associated with increasing temperature. The effect of ambient temperature exposure appears to be modified by sex and age, with males aged between 16 and 64 years being the most vulnerable population. When we assessed the change in magnitude of this effect over time, we found that the temperature-related asthma hospitalisation risk is largest during 2002–2007, whereas there is weak, if any, effect during 2008–2013 and 2014–2019. We also observed some spatial variation with populations living in the regions of Yorkshire and the Humber and East and West Midlands being most vulnerable.

Our study is comparable with previous studies assessing asthma hospital admissions and ambient temperature during warmer months, rather than studies assessing heat waves and extreme temperatures.^8^ Our study is in line with a time series study in Hong Kong using lag 0–3 mean temperature and accounting for relative humidity, wind speed, solar radiation, influenza and air pollution, which reported an increased asthma hospitalisation relative risk (1.19; 95% CI 1.06, 1.36) at 30°C compared with 27°C during the hot season.^15^ The same study reported that the effect of temperature during the hot season was largest in adults aged between 15 and 59 years.^15^ Our results are in contrast with those of a time series study in Shanghai, China, focusing on different temperature metrics and accounting for meteorology and air pollution, which reported no association between asthma hospitalisations and warm temperatures.^10^ A case–crossover study in Brazil during 2000–2015 examined temperature variability and asthma hospitalisations and reported a higher effect in people aged 80 or older, with weak evidence of effect modification by sex.^16^ In our study, there is weak evidence that people aged 65 or older were vulnerable, while we observed a strong effect modification by sex for temperature-related asthma hospitalisations.

The age modification we observed might have multiple explanations. People aged between 5 and 64 years are likely to be exposed more to environmental triggers in comparison to the older subgroup who stay mostly at home, do not work and have a less active social life. The adult population could also be more vulnerable due to the higher prevalence of smoking in the adult population resulting in a more severe airway inflammation and lower lung function, which potentially could lead to increased vulnerability to higher temperatures.^15 37^ The above suggests engaging patients and carers to raise awareness of the adverse effects of temperature. The weak observed effect in the older population could also reflect outcome misclassification (non-differential) due to the multiple comorbidities that this population is suffering from.

We observed that the effect of ambient temperature on asthma hospitalisations has attenuated over time. Potential explanations of this trend include adaptive mechanisms to heat exposure over time,^21^ such as more consistent adherence to medication over time, effectiveness of the heat alerts (they were introduced in 2004), infrastructure changes and improved healthcare. Based on the period analysis, the effect is most prominent in males aged 15–65 years, which could also point towards sex-related differences in exposure due to differences in behaviours, lifestyle and occupation, but also could imply differences in the prevalence of other comorbid chronic diseases, such as chronic obstructive pulmonary disease.^38^ Nevertheless, we cannot rule out potential residual confounding due to data availability or due to differences in the exposure (duration of heat period, changes in maximum/minimum temperatures) across the years. The decreasing trend should be further monitored and re-examined in the future considering future changes in the climate, factors influenced by these changes (eg, allergen concentration) and the increasing burden of asthma.

In line with our study, previous studies in Taiwan and Brazil have reported effect modification by space.^11 16^ We observed that populations in the regions of Yorkshire and the Humber and East Midlands are the most vulnerable. Potential explanations include spatial effect modifiers that are most prominent in these regions, such as allergen concentration, higher air pollution levels (particulate matter) and higher prevalence of smoking. Nevertheless, we should iterate that the evidence for heterogeneity in the effect by space is weak as the CrIs of the effects overlap with the CrIs of the effects nationwide.

The observed discrepancies with some previous studies can have multiple explanations. The choice of confounding adjustment can impact the results. In addition, the temperature metrics and lags used across the studies are not consistent. Apart from min, mean, max and range of temperature, a couple of studies have used temperature variation.^16 39^ In our study we used the mean, as we were interested in the overall effect of the temperature during the day and were not interested in the impact of night temperatures (using min) or extreme heat during midday (using max). It is hard, nevertheless, to predict how this choice could impact the results, as the different temperature metrics point towards different exposure windows. Previous studies had available coarser geographical resolution (city or county level), which can lead to misclassification of the exposure and inadequate confounding adjustment. In addition, most previous studies are focused on urban settings,^9 12 15–17^ whereas our study provides nationwide estimates. Last, resources for healthcare setting, health promotion and infrastructure across the different countries could contribute to the observed discrepancies.

The main strength of our study is the availability of high geographical resolution of the outcome. This allows us to link the outcome with the exposure and confounders with very high precision minimising potential misclassification. As hospitalisations were retrieved from NHS Digital (nationwide central data on hospital admissions), we expect to cover most of the asthma hospitalisation burden in England, minimising any selection bias. We used individual-level data, which allows us to examine individual-level vulnerabilities, for example, related to age and sex, and to avoid ecological bias.

We assigned temperature exposure using the residential address of the cases and used this as a proxy for individual exposure to temperature. This proxy is expected to be more accurate for adults aged 65 years or older who are expected to stay longer at home or somewhere in the direct proximity. In addition to this, we used the outdoor temperature which is expected to be different from the temperature inside the house. The misclassification induced by this is not expected to be differential and to lead to bias. Data for relative humidity and wind speed were only available at coarse geographical resolution, namely at 11 km×11 km, which likely misses the localised trends in these covariates and could potentially lead to inadequate adjustment for confounding. The results depend on the assumption that air pollution is a mediator and not a confounder. We could not check the validity of this assumption, as data for air pollution are available only after 2008, when the effect of temperature on asthma hospital admissions is weak.

The direct biological mechanism by which higher temperatures trigger asthma exacerbations is unclear. Warm temperatures can aggravate the respiratory system-related burden, as they can affect the electrolyte balance.^40^ High temperatures may activate the c-fibre nerve and enhance bronchoconstriction leading to higher morbidity.^15^ In addition, warmer temperatures can increase allergen or air pollution (in particular O_3_) concentrations, or help the reproduction and spread of viruses and bacteria causing or aggravating respiratory diseases.^15^

This study provides evidence of an association between warm temperatures and asthma exacerbations in England. More studies in counties with different climate, healthcare and social behaviours are needed to further understand the generalisability of the results. The effect of warm temperatures on asthma hospitalisation has attenuated over time suggesting potential adaptive mechanisms to heat exposure or differences in behaviours, lifestyle, comorbid conditions, other environmental exposures and occupation over time.

## Data Availability

Data may be obtained from a third party and are not publicly available. The Small Area Health Statistics Unit (SAHSU) does not have permission to supply data to third parties. For reproducibility purposes we have simulated data and provided the code used for the analysis at https://github.com/gkonstantinoudis/asthma_temperature.
