# Supplementary material for: Asthma hospitalisations and heat exposure in England: a case–crossover study during 2002–2019
Source: Thorax. 2023 Apr 17;78(9):875–81. doi: 10.1136/thorax-2022-219901 (PMC10447396; doi:10.1136/thorax-2022-219901)
Supplement: Supplementary data [file thorax-2022-219901supp001.pdf]

Online Supplement: "Asthma hospitalisations and heat exposure in England: A case-crossover study during 2002-2019."

Garyfallos Konstantinoudis[1,*], Cosetta Minelli[2], Holly Ching-yu Lam[3], Elaine Fuertes[2], Joan Ballester[4], Bethan Davies[1,5], Ana Maria Vicedo Cabrera[6,7], Antonio Gasparrini[8,9,10], and Marta Blangiardo[1]

[1] *MRC Centre for Environment and Health, Department of Epidemiology and Biostatistics, School of Public Health, Imperial College London, London, UK*

[2] *National Heart and Lung Institute, Imperial College London, London, United Kingdom*

[3] *UK Health Security Agency, UK*

[4] *ISGlobal, Barcelona, Spain*

[5] *UK Small Area Health Statistics Unit, Imperial College London, London, UK*

[6] *Oeschger Center for Climate Change Research, University of Bern, Bern, Switzerland*

[7] *Institute of Social and Preventive Medicine, University of Bern, Switzerland*

[8] *Department of Public Health Environments and Society, London School of Hygiene Tropical Medicine, London, UK*

[9] *Centre for Statistical Methodology, London School of Hygiene Tropical Medicine, London, UK*

[10] *Centre on Climate Change and Planetary Health, London School of Hygiene Tropical Medicine, London, UK*

[*] Corresponding author. Email: g.konstantinoudis@imperial.ac.uk

Konstantinoudis G, *et al. Thorax* 2023; 78:875–881. doi: 10.1136/thorax-2022-219901

Contents

S1 Text **3**

S1.1 Model description 3

List of Tables

S1 Number and proportion of hospital records by age, sex and period. 4

S2 Percentage hospitalisation risk for every 1°C increase in the temperature and 95% credible intervals by sex and age for the unadjusted and fully adjusted (precipitation and national holidays) models. 5

S3 Percentage hospitalisation risk for every 1°C increase in the temperature and 95% credible intervals by sex, age and period for the fully adjusted (precipitation, relative humidity, wind speed, national holidays and recurrent hospitalisations) model. 6

List of Figures

S1 Directed acyclic graph (DAG) for the association between temperature and asthma hospitalisations. The DAG does not show confounders that are accounted for through the case-cross over study design, for instance urbanicity and socio-economic deprivation but also day of the week, seasonality and long-term trends that are accounted through the sampling. The box with age, sex, time, and space denotes effect modifiers and not confounders. The DAG does not provide an exhaustive list of the potential effect modifiers of this relationship. 7

S2 Regions in England. 8

S3 Flowchart of the population. 9

S4 Random walks of order 2 on the hospitalisation relative risk by age and sex to allow flexible fits in the unadjusted and adjusted (precipitation, relative humidity, wind speed, national holidays and recurrent hospitalisations) models. The hospitalisation relative risk is relative to the risk at 15°C. 10

S5 Percentage hospitalisation risk for every 1°C increase in the temperature and 95% credible intervals for the fully adjusted (precipitation and national holidays) model across the 0-5 lags. 11

Konstantinoudis G, *et al. Thorax* 2023; 78:875–881. doi: 10.1136/thorax-2022-219901

S1 Text

S1.1 Model description

Let Y_{mjk} be the case-control identifier for the asthma hospitalisation for the event (case or control) at the m grid cell and day, in the j-th case-control group and k-th patient. Let also X_m be the temperature at m grid cell and day and $Z_m = (1, Z_{1m}, Z_{2m})$ a vector denoting the different confounders. Then:

$$Y_{mjk} \sim \text{Poisson}(\mu_{mjk})$$

$$\log(\mu_{mjk}) = f(X_m) + \alpha Z_m + u_j + w_k$$

$$w_k \sim N(0, \sigma_1^2)$$

In the main analysis we set $f(X_m) = \beta X_m$ whereas for the sensitivity analysis $f(\cdot)$ is the non-linear effect of the m daily temperature in each grid cell. To complete the prior specification in the above model, we specify priors for terms $\alpha, \beta \sim N(0, 1000)$ and $u_j \sim N(0, 100)$.

To define the non-linear effect for the sensitivity analysis, we assume the following second-order random walk (RW2) model:

$$X_{im} \mid X_{(i-1)m}, X_{(i-2)m}, \tau_X \sim \text{Normal}\left(2X_{(i-1)m} + X_{(i-2)m}, \tau_X^{-1}\right), \quad (1)$$

with σ_x denoting the standard deviation. For the standard deviation σ_x we selected a penalised complexity prior so that $\Pr(\sigma_x > 1) = 0.01$ [1]. This prior penalises complexity from the null model, i.e. gives enough mass to 0, reflecting our scepticism that recurrent hospitalisation will affect the observed relationship.

Conditioning on the fixed effect of the case-control group u_j, a Poisson model provides a flexible alternative to the conditional logistic regression for case-cross over analysis [2]. We added the parameter w_k to account for patient clustering due to exacerbation history. We fit the above model for the different age and sex groups for the different period and regions.

Konstantinoudis G, *et al. Thorax* 2023; 78:875–881. doi: 10.1136/thorax-2022-219901

Tables

Table S1: Number and proportion of hospital records by age, sex and period.

Age	Total	Females	2002-2007	2008-2013	2014-2019
5-15	50,516 (23%)	19,915 (24%)	15,768 (22%)	16,740 (15%)	18,008 (23%)
16-64	135,011 (61%)	89,336 (61%)	42,305 (62%)	41,774 (67%)	50,932 (61%)
64>	34,765 (17%)	23,764 (15%)	11,730 (16%)	10,382 (18%)	12,653 (16%)
Total	220,292 (100%)	133,015 (100%)	69,803 (100%)	68,896 (100%)	81,593 (100%)

Konstantinoudis G, *et al. Thorax* 2023; 78:875–881. doi: 10.1136/thorax-2022-219901

Table S2: Percentage hospitalisation risk for every 1°C increase in the temperature and 95% credible intervals by sex and age for the unadjusted and fully adjusted (precipitation and national holidays) models.

Age	Sex	Unadjusted	Adjusted
5-15	Male	1.30 (0.75, 1.85)	1.44 (0.83, 2.05)
5-15	Female	0.37 (-0.31, 1.06)	0.63 (-0.12, 1.39)
5-15	Total	0.95 (0.52, 1.38)	1.16 (0.69, 1.64)
16-64	Male	1.86 (1.40, 2.32)	2.10 (1.59, 2.61)
16-64	Female	1.19 (0.86, 1.51)	0.98 (0.62, 1.34)
16-64	Total	1.42 (1.15, 1.69)	1.38 (1.09, 1.68)
64>	Male	0.32 (-0.61, 1.24)	0.16 (-0.85, 1.17)
64>	Female	0.18 (-0.45, 0.81)	-0.24 (-0.92, 0.45)
64>	Total	0.24 (-0.28, 0.76)	-0.08 (-0.65, 0.49)
Total	Male	1.48 (1.15, 1.81)	1.66 (1.30, 2.03)
Total	Female	0.89 (0.63, 1.16)	0.73 (0.43, 1.02)
Total	Total	1.13 (0.92, 1.34)	1.11 (0.88, 1.34)

Table S3: Percentage hospitalisation risk for every 1°C increase in the temperature and 95% credible intervals by sex, age and period for the fully adjusted (precipitation, relative humidity, wind speed, national holidays and recurrent hospitalisations) model.

Age	Sex	2002 - 2007	2008 - 2013	2014 - 2019
5-15	Male	3.06 (2.01, 4.13)	0.55 (-0.53, 1.64)	-0.45 (-1.47, 0.59)
5-15	Female	1.14 (-0.22, 2.51)	-0.31 (-1.63, 1.01)	0.31 (-0.93, 1.55)
5-15	Total	2.48 (1.64, 3.33)	0.30 (-0.54, 1.14)	-0.08 (-0.88, 0.72)
16-64	Male	4.58 (3.71, 5.46)	-0.24 (-1.16, 0.69)	0.67 (-0.17, 1.52)
16-64	Female	2.90 (2.24, 3.56)	-0.09 (-0.74, 0.56)	-0.06 (-0.64, 0.52)
16-64	Total	3.59 (3.07, 4.12)	-0.12 (-0.65, 0.42)	0.21 (-0.27, 0.69)
64>	Male	0.54 (-1.13, 2.23)	-0.65 (-2.54, 1.27)	-0.34 (-1.98, 1.32)
64>	Female	1.13 (-0.03, 2.32)	-0.58 (-1.83, 0.69)	-1.48 (-2.61, -0.33)
64>	Total	1.05 (0.08, 2.02)	-0.50 (-1.55, 0.57)	-1.03 (-1.97, -0.08)
Total	Male	3.67 (3.04, 4.30)	0.10 (-0.56, 0.77)	0.25 (-0.36, 0.87)
Total	Female	2.37 (1.84, 2.90)	-0.17 (-0.70, 0.37)	-0.20 (-0.68, 0.28)
Total	Total	2.96 (2.56, 3.37)	-0.04 (-0.46, 0.37)	-0.01 (-0.39, 0.37)

Konstantinoudis G, *et al. Thorax* 2023; 78:875–881. doi: 10.1136/thorax-2022-219901

Figures

Figure S1: Directed acyclic graph (DAG) for the association between temperature and asthma hospitalisations. The DAG does not show confounders that are accounted for through the case-cross over study design, for instance urbanicity and socio-economic deprivation but also day of the week, seasonality and long-term trends that are accounted through the sampling. The box with age, sex, time, and space denotes effect modifiers and not confounders. The DAG does not provide an exhaustive list of the potential effect modifiers of this relationship.

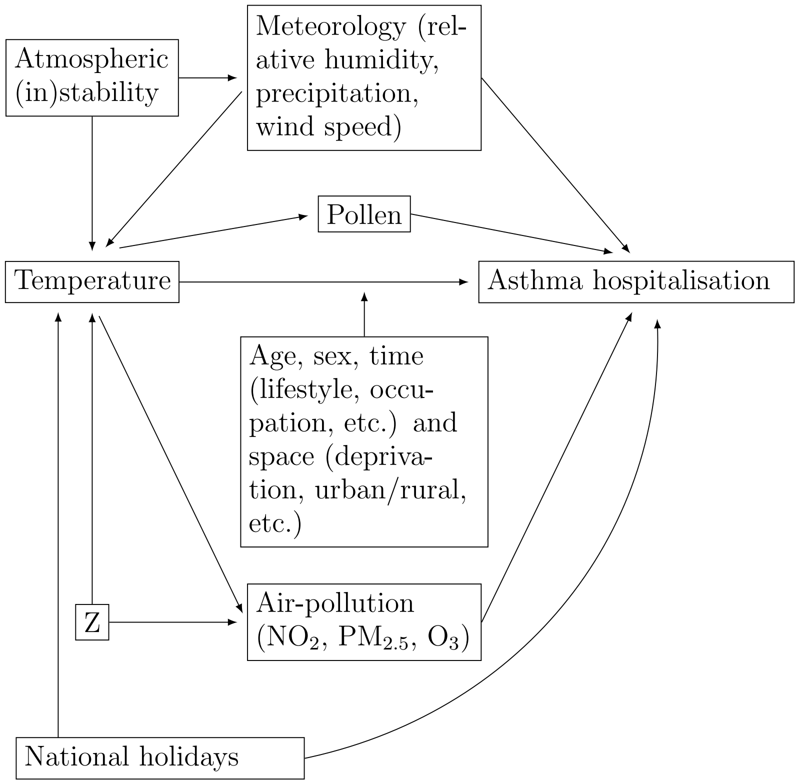

Konstantinoudis G, *et al. Thorax* 2023; 78:875–881. doi: 10.1136/thorax-2022-219901

Figure S2: Regions in England.

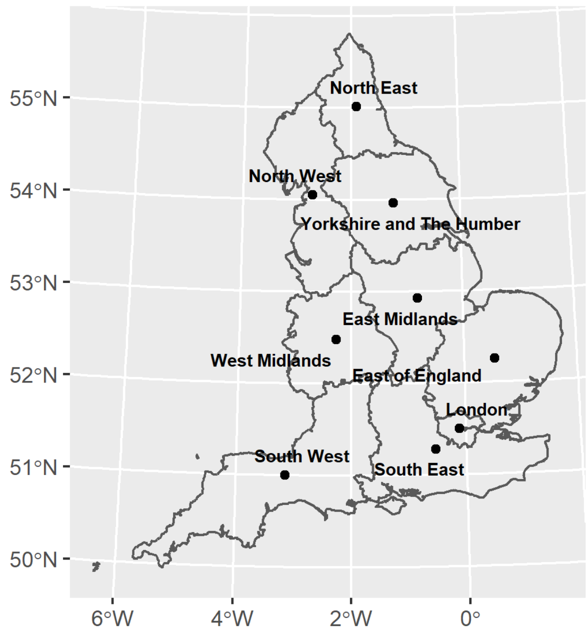

Konstantinoudis G, *et al. Thorax* 2023; 78:875–881. doi: 10.1136/thorax-2022-219901

BMJ Publishing Group Limited (BMJ) disclaims all liability and responsibility arising from any reliance placed on this supplemental material which has been supplied by the author(s)

Figure S3: Flowchart of the population.

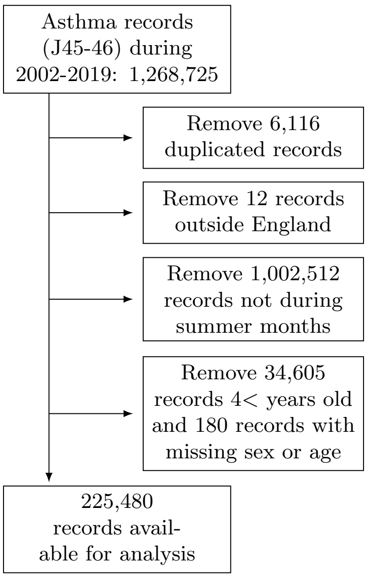

Konstantinoudis G, *et al. Thorax* 2023; 78:875–881. doi: 10.1136/thorax-2022-219901

Figure S4: Random walks of order 2 on the hospitalisation relative risk by age and sex to allow flexible fits in the unadjusted and adjusted (precipitation, relative humidity, wind speed, national holidays and recurrent hospitalisations) models. The hospitalisation relative risk is relative to the risk at 15°C.

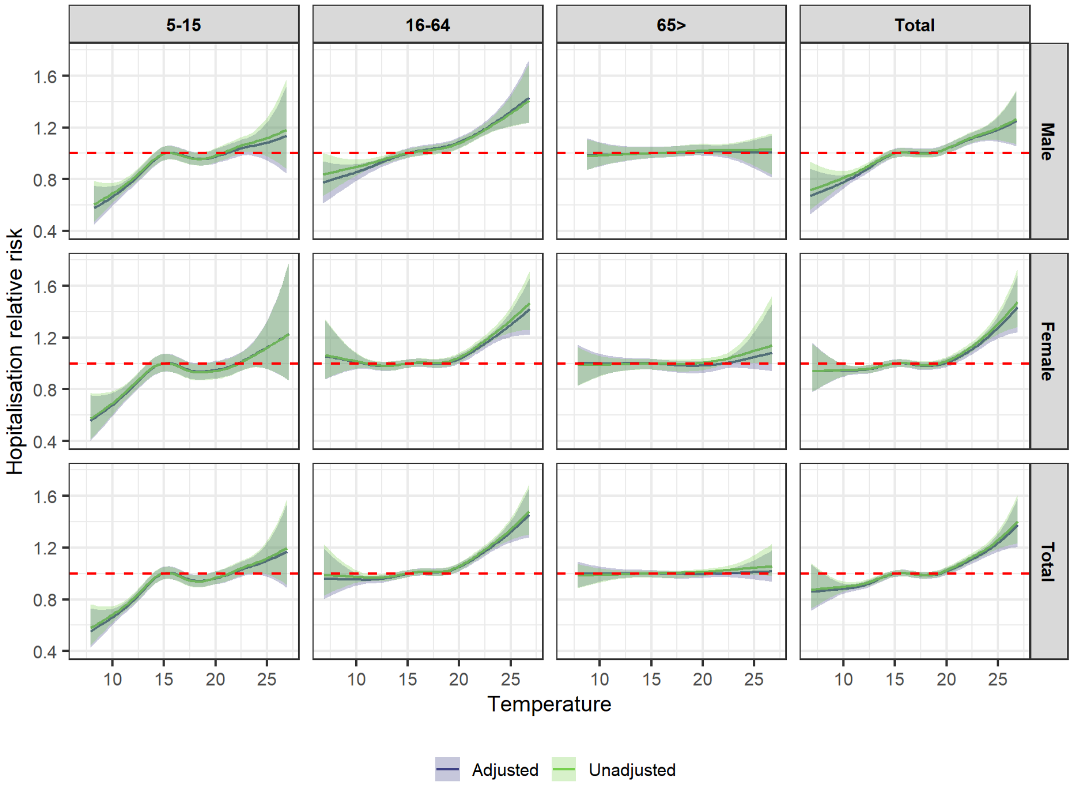

Konstantinoudis G, *et al. Thorax* 2023; 78:875–881. doi: 10.1136/thorax-2022-219901

Figure S5: Percentage hospitalisation risk for every 1°C increase in the temperature and 95% credible intervals for the fully adjusted (precipitation and national holidays) model across the 0-5 lags.

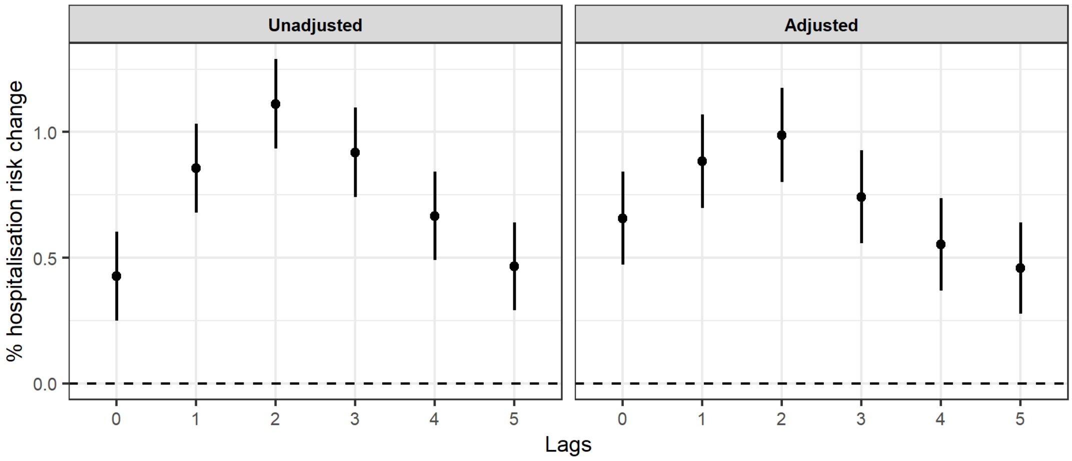

Konstantinoudis G, *et al. Thorax* 2023; 78:875–881. doi: 10.1136/thorax-2022-219901

References

[1] Daniel Simpson, Håvard Rue, Andrea Riebler, Thiago G Martins, and Sigrunn H Sørbye. Penalising model component complexity: A principled, practical approach to constructing priors. *Statistical science*, 32(1):1–28, 2017.

[2] Ben G Armstrong, Antonio Gasparrini, and Aurelio Tobias. Conditional poisson models: a flexible alternative to conditional logistic case cross-over analysis. *BMC medical research methodology*, 14(1):1–6, 2014.

Konstantinoudis G, *et al. Thorax* 2023; 78:875–881. doi: 10.1136/thorax-2022-219901
